# The terrein biosynthetic gene cluster of *Aspergillus terreus*: structure, function, regulation, and similar gene clusters

**DOI:** 10.3389/ffunb.2025.1696451

**Published:** 2026-01-12

**Authors:** Márk Z. Németh, Sándor Csíkos, Gábor M. Kovács

**Affiliations:** 1 Department of Plant Anatomy, Institute of Biology, ELTE Eötvös Loránd University, Budapest, Hungary; 2 Plant Protection Institute, HUN-REN Centre for Agricultural Research, Budapest, Hungary

**Keywords:** 6-hydroxymellein, epigenetic regulation, gene knockout, overexpression, polyketide, secondary metabolite, transcriptional regulation

## Abstract

Fungi synthesize a wide variety of secondary metabolites (SMs). The genes of the biosynthetic pathways of many of these compounds are encoded by biosynthetic gene clusters (BGCs), which typically consist of a core biosynthetic enzyme, tailoring enzymes, transporters, and pathway-specific regulators. One of the well-studied fungal SMs is the polyketide terrein, which is produced by *Aspergillus terreus* and exhibits a wide range of biological activities, such as cytotoxic, phytotoxic, and antibacterial effects. The structure and function of the terrein BGC, the functions of the encoded proteins, and the processes controlling the transcriptional regulation of the BGC are summarized in this mini review. Both pathway-specific and global regulators and epigenetic regulation are presented. Furthermore, similar BGCs identified in other fungal taxa are introduced in short. Despite significant advances, key aspects of terrein biosynthesis, such as some protein functions, details of the BGC regulation, and SM ecological functions remain unresolved. Filling in these gaps will help us better understand the biology of fungal SMs and could pave the way for biotechnological applications.

## 1 Introduction

Fungi produce a plethora of secondary metabolites (SMs) (Macheleidt et al., 2016). These are structurally diverse, low-molecular-mass compounds that are not essential for growth under standard conditions (Brakhage, 2013). These metabolites are characterized by a huge functional diversity, such as communication, competition and pathogenicity, among others (Macheleidt et al., 2016).

The genes responsible for the biosynthesis of several SMs are often encoded by secondary metabolic clusters (Rokas et al., 2018), also called biosynthetic gene clusters (BGCs) (Keller, 2019). These clusters typically follow a common organizational pattern (Rokas et al., 2018). They usually include a core gene encoding an enzyme, such as a nonribosomal peptide synthetase, polyketide synthase (PKS), dimethylallyl tryptophan synthetase or terpene cyclase. The core enzyme produces the precursor, or backbone of the SM(s). In addition, BGCs typically contain genes encoding tailoring enzymes that modify the precursor compound, transporters that facilitate the export of the final metabolite, and a transcription factor that regulates the expression of the cluster genes. These latter genes, however, may be missing from the BGC, and occasionally, hypothetical or enigmatic genes are also present in the cluster (Keller, 2019).

**Figure 1:**
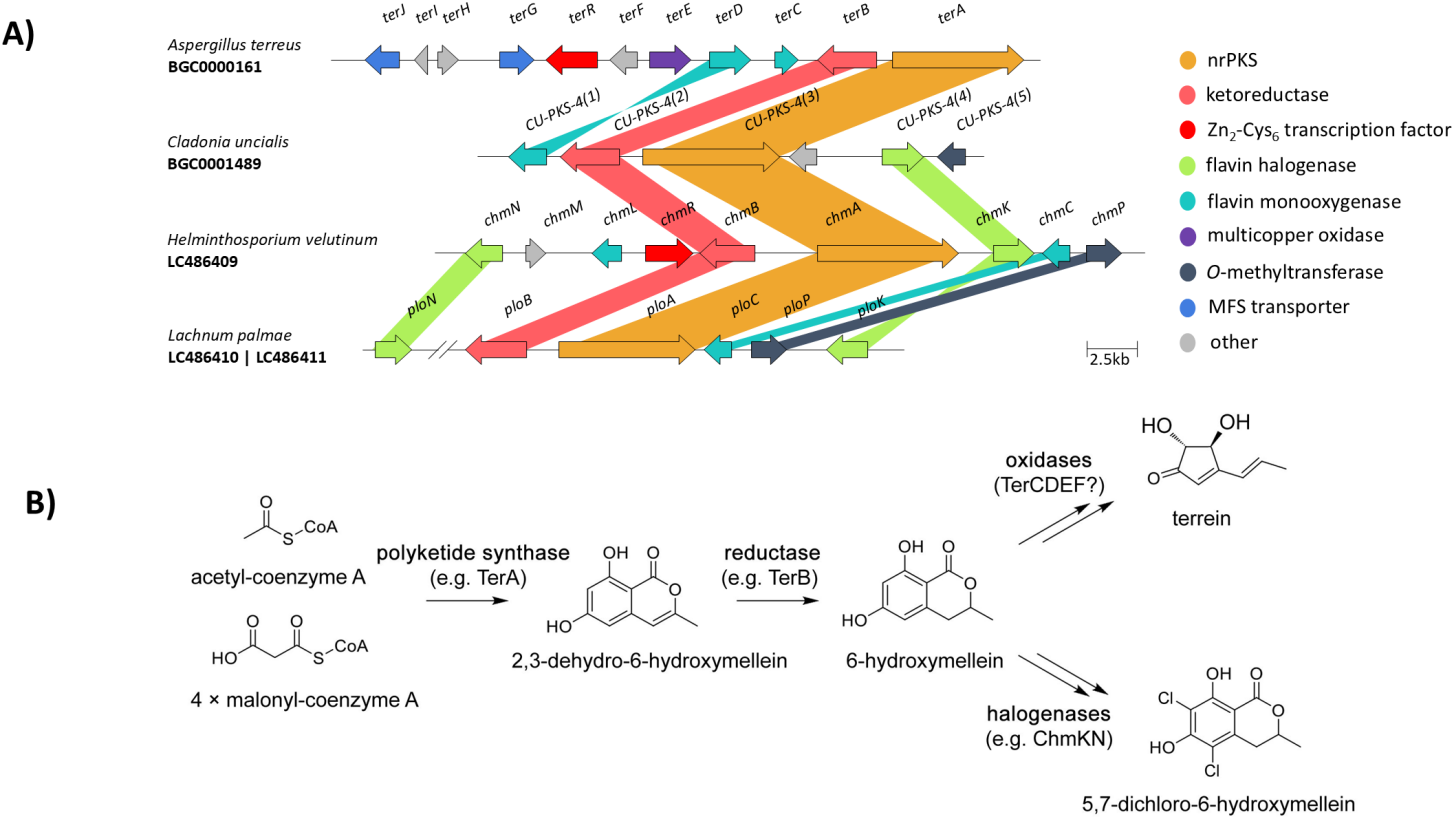
**(A)** The structure of the *ter* BGC of *A. terreus*, and similar biosynthetic gene clusters in other organisms. Comparison was made using the clinker (Gilchrist and Chooi, 2021) module of CAGECAT (van den Belt et al., 2023) with 0.3 identity threshold. Color coding refers to predicted functions, and putative homologous genes are connected by stripes. **(B)** The schematic biosynthetic pathway of terrein and other, related metabolites deriving from 6-hydroxymellein. nrPKS, non-reducing polyketide synthase; MFS, major facilitator superfamily.

The polyketide terrein (**Figure 1**), produced by the filamentous fungus *Aspergillus terreus* (Yin et al., 2016), is among the better studied fungal SMs. Terrein has been shown to have antibacterial, antifungal (Gressler et al., 2015b), anti-inflammatory, antioxidative (Lee et al., 2010), antiproliferative, cytotoxic (Liao et al., 2012) and proapoptotic (Demasi et al., 2010; Porameesanaporn et al., 2013) properties, among others. In addition, it has phytotoxic activity, as it causes lesions on fruit surfaces, and inhibits plant seed germination (Zaehle et al., 2014). Furthermore, it is also used by the fungus as a reductive agent, reducing ferric ions to ferrous iron ions, increasing iron solubility and facilitating iron uptake (Gressler et al., 2015b). These latter may be the most important functions fulfilled in the ecological context in *A. terreus* (Zaehle et al., 2014; Gressler et al., 2015b).

The relatively simple chemical structure (Yin et al., 2016), the ability to produce it in high yields (Yao et al., 2022) and the emerging insights into the regulation of its synthesis (see below) make terrein a promising model candidate for studying fungal secondary metabolism. Accordingly, the BGC coding the genes needed for the biosynthesis of terrein (termed *ter* BGC in the present work (Kahlert et al., 2021b)) may serve as a representative system for investigating the structure and regulation of fungal SM gene clusters.

This mini review aims to summarize current knowledge on the structure and regulation of the terrein biosynthetic gene cluster, and to introduce the similar gene clusters present in other organisms.

## 2 Structure of the terrein biosynthetic gene cluster

The *ter* BGC was identified during a search for a non-reducing PKS potentially responsible for the coloration of conidia in *A. terreus*, which led to the identification of a PKS, encoded by the gene *ATEG_00145* (Zaehle et al., 2014). The elimination of the gene did not change the coloration of the conidia; however, metabolic profiling showed that a metabolite, later identified as terrein, was completely absent from the culture broth of the mutant (Zaehle et al., 2014). Based on these results, the PKS encoded by the gene *ATEG_00145* (or *terA*) was denoted TerA, referring to the SM terrein (Zaehle et al., 2014).

Starting from the *terA* locus, upstream and downstream neighboring genes were investigated whether they were coexpressed with *terA*. Eleven genes from *ATEG_00135* to *ATEG_00144* were found to be coexpressed with the PKS encoding gene, and thus were considered to belong to a single BGC (Zaehle et al., 2014). Starting from *ATEG_00144*, (right upstream of *terA*), the genes were further labeled from *terB* (*ATEG_00144*) to *terJ* (*ATEG_00135*). A gene (*ATEG_00139*), coding for a transcription factor, the putative regulator of the cluster, was denoted *terR* (Zaehle et al., 2014). Some of the genes of the BGC are encoded on the forward strand and others on the reverse strand (**Table 1**) (Zaehle et al., 2014). The approx. 43.5 kbp long *ter* BGC has also been deposited in the Minimum Information about a Biosynthetic Gene cluster (MIBiG) database, a reference database and repository for BGCs (Zdouc et al., 2025) as BGC0000161 (**Figure 1**).

**Table 1:** List of genes constituting the *Aspergillus terreus* terrein biosynthetic gene cluster, and the functions of the encoded proteins (Zaehle et al., 2014).

Locus tag	Simplified gene name	Orientation relative to *terA*	Protein function
*ATEG_00135*	*terJ*	reverse	membrane transport protein belonging to the major facilitator superfamily
*ATEG_00136*	*terI*	reverse	glyoxalase, dioxygenase
*ATEG_00137*	*terH*	forward	NAD-binding epimerase
*ATEG_00138*	*terG*	forward	membrane transport protein belonging to the major facilitator superfamily
*ATEG_00139*	*terR*	reverse	Zn_2_Cys_6_ transcription factor
*ATEG_00140*	*terF*	reverse	Kelch domain containing oxidase
*ATEG_00141*	*terE*	forward	multicopper oxidase
*ATEG_00142*	*terD*	forward	FAD-dependent monooxygenase
*ATEG_00143*	*terC*	forward	FAD-dependent monooxygenase
*ATEG_00144*	*terB*	reverse	multidomain dehydratase - ketoreductase
*ATEG_00145*	*terA*	forward	non-reducing polyketide synthase

As a whole, the organization of the *ter* BGC reflects a typical compact fungal PKS cluster with a core enzyme, additional genes, and a cluster-specific regulator.

## 3 Functions of proteins encoded in the terrein biosynthetic gene cluster

The general molecular functions of the proteins encoded in the *ter* BGC are shown in **Table 1**. The non-reducing polyketide synthase TerA is responsible for the synthesis of the precursors of terrein. The protein utilizes acetyl-CoA as a starter, and extends the fatty acid chain using malonyl-CoA units (Zaehle et al., 2014). The low extension cycle specificity enables adding two to four extender units, leading to different chain length products (orsellinic acid, 4-hydroxy-6-methylpyrone and 2,3-dehydro-6-hydroxymellein) (Zaehle et al., 2014). TerB reduces 2,3-dehydro-6-hydroxymellein to 6-hydroxymellein (**Figure 1**). Then, the four malonyl-CoA-derived 6-hydroxymellein serves as a precursor to terrein (Yao et al., 2022) and, potentially, to several other SMs (Ugai et al., 2020; Kahlert et al., 2021b) (**Figure 1**; and see below). TerA and TerB act collaboratively, and a close interaction between the two was proposed (Kahlert et al., 2021b).

The flavin-dependent monooxygenase TerC catalyzes the decarboxylation of 6-hydroxymellein. The resulting intermediate is hydroxylated by TerD, another flavin-dependent monooxygenase (Kahlert et al., 2021a). TerE is also an oxidase, and potentially, TerF is as well (Yin et al., 2016). However, the exact functions of these oxidases are yet to be determined (Zaehle et al., 2014; Yin et al., 2016).

The function of the two major facilitator superfamily transporters TerG and TerJ, is yet unknown; however, they may be responsible for the terrein secretion (Zaehle et al., 2014), possibly also providing self-resistance to the metabolite (Keller, 2015), or they may be not directly and strictly involved in the terrein biosynthesis.

TerH is predicted to be an epimerase, and TerI is a predicted glyoxalase and dioxygenase. Deletion mutants of *terH* and *terI* produce terrein in lower amounts than the wild type, showing that these genes may influence, but are not essential for terrein biosynthesis (Zaehle et al., 2014). The exact functions are not known (Yin et al., 2016). Finally, TerR is a zinc-binding Zn_2_Cys_6_ transcription factor, regulating the transcription of the other genes in the cluster (see below).

Taken together, the proteins encoded in the *ter* BGC convert simple precursors into terrein through a sequence of polyketide synthesis and tailoring reactions, and presumably also contribute to its secretion.

## 4 Regulation of the terrein biosynthetic gene cluster

The control of fungal secondary metabolism involves multiple layers of regulation, such as pathway-specific factors directly regulating transcription, global regulatory proteins exerting indirect regulation, and epigenetic mechanisms (Macheleidt et al., 2016).

### 4.1 Direct transcriptional regulation

TerR is an essential, direct regulator of the *ter* gene cluster. Disruption of *terR* results in lack of terrein and other, related SMs (Zaehle et al., 2014). TerR belongs to the group of transcriptional activators with a GAL4-type Zn_2_Cys_6_ zinc binuclear cluster DNA-binding domain (Gressler et al., 2015a). Transcription factors with this domain are commonly found in fungi, and the domain is the most common type of DNA-binding domains in transcription factors regulating BGCs (MacPherson et al., 2006). The N-terminal of the protein is essential for DNA binding (Gressler et al., 2015a).

All genes of the *ter* BGC are expressed in the fungus in inducing conditions. In the *terR* knockout (KO) mutants *terE*, *terF* and *terH* remain expressed at some level, which is considered a background expression (Gressler et al., 2015a), indicating that the expression of these genes is not solely dependent on TerR. On the other hand, the presence of TerR alone is sufficient for the cluster activation, meaning that no other factors or activators are needed, as when *terR* was expressed in a heterologous system, *A. niger*, terrein was produced (Gressler et al., 2015a).

*TerA* and *terB* are transcribed in opposite directions within the terrein *ter* BGC (**Table 1**, **Figure 1**) (Zaehle et al., 2014). These two genes share a common bidirectional promoter (Gressler et al., 2015a). The expression levels are different tough, the expression of *terA* being 8–14-fold higher than that of *terB*. *TerE* and *terF*, as well as *terH* and *terI*, are also arranged in opposite directions and possess comparable bidirectional promoters (Gressler et al., 2015a). Interestingly, TerR has been shown to exist as a monomer, nevertheless, the promoter has also been reported to enable the binding of a second TerR protein (Gressler et al., 2015a). The consensus sequence for high-affinity TerR binding sites is 5′−TCGGHHWYHCGG−3′. At least one of the high-affinity motifs is present in the promoters of most genes needed for terrein synthesis (*terA-F* and *terJ*), but were not detected in the promoter regions of *terG*, *terH*, and *terI*, which are dispensable for terrein production (Gressler et al., 2015a).

Experiments have also shown that the expression rate of the *ter* BGC is directly regulated by the amounts of TerR, and that TerR levels are the rate limiting step in the promoter activation (Gressler et al., 2015a). The number of motifs and their distance from the transcription start site appear to influence the strength of transcriptional activation (Gressler et al., 2015a).

In summary, TerR is the central activator of the *ter* BGC. Several genes in the cluster share bidirectional promoters, and number of TerR-binding motifs determine transcription intensity. Most essential genes contain high-affinity TerR motifs, whereas dispensable genes do not.

### 4.2 Signals influencing terrein production

Experiments have shown that terrein production is induced when *A. terreus* is inoculated onto fruits (Gressler et al., 2015b). Accordingly, one of conducive conditions for terrein production is growth on sugar-rich plant-derived media such as potato dextrose broth (PDB) (Zaehle et al., 2014). Complex media, such as banana or apple juice cause even stronger gene expression, exceeding that of the expression in PDB. Glucose minimal medium, however, is not permissive for the terrein synthesis. Potato broth does not induce the expression of genes in the cluster by itself, only in the presence of glucose. This points that glucose is required for terrein production (Gressler et al., 2015b).

Growing the fungus on glucose minimal medium supplemented with different additives revealed that methionine triggers a significant induction even at low concentrations (Gressler et al., 2015b). The same was shown for iron limitation (Gressler et al., 2015b). Furthermore, nitrogen starvation is another, strong inductor of BGC activation, and it is considered as one of the major triggers in natural conditions (Gressler et al., 2015b).

Overall, terrein synthesis is promoted by glucose-containing and plant-derived media, while simple glucose minimal medium alone does not induce BGC activation. Specific signals, such as methionine, iron limitation, and nitrogen starvation strongly induce terrein biosynthesis.

### 4.3 Indirect transcriptional regulation

Terrein production occurs only under certain environmental conditions, implying that *terR* transcription and activation are regulated by additional transcription factors that transmit environmental signals to the terrein biosynthetic pathway (Gressler et al., 2015a). The signal transduction pathways, which transform environmental stimuli into changes in secondary metabolism (Macheleidt et al., 2016) and their potential crosstalk (Brakhage, 2013) has yet to be examined in this model system.

Some of the downstream global regulators are, however, well characterized. The global transcription factors AreA and AtfA are essential for *ter* BGC induction during nitrogen starvation (Gressler et al., 2015b). AreA is a global nitrogen regulator (Davis et al., 2005), and AtfA is a stress response transcription factor (Balázs et al., 2010). Accordingly, the elimination of any of the genes encoding these two transcription factors results in strongly impaired terrein production in nitrogen limited conditions, and the double mutant completely lacks terrein (Gressler et al., 2015b). Overexpression of *atfA* causes increased terrein production, which proves the involvement of the protein in *ter* BGC induction. *AtfA* is suspected to be induced by a methionine-dependent signaling cascade, and AtfA induction then leads to *terR* expression (Gressler et al., 2015b). Indeed, two sites matching the consensus sequence of AreA binding sites can be found in the promoter region of *terR*, suggesting a direct role of AreA in the activation of *terR* expression, and consequently, the activation of the *ter* BGC (Gressler et al., 2015b).

HapX is a transcriptional inducer under iron limitations (Hortschansky et al., 2007). The *hapX* gene has been shown to influence the activation of the *ter* BGC, as its elimination significantly reduced the amount of terrein produced (Gressler et al., 2015b). Accordingly, a putative HapX binding site is suspected in the promoter of *terR* (Gressler et al., 2015b).

Taken together, terrein production depends on environmental cues, indicating that terR is controlled by upstream regulators that have not yet been fully explored in this system. The global regulators AreA and AtfA are key activators of the *ter* BGC during nitrogen starvation and methionine-triggered signaling, directly promoting terR expression. Under iron limitation, HapX also contributes to *ter* BGC activation.

### 4.4 Epigenetic regulation

Specific studies on epigenetic regulation of terrein biosynthesis in *A. terreus* are limited. Elimination of the *hstD*, a fungal-specific histone deacetylase gene from *A. terreus* caused significant upregulation of *ter* gene expression, and consistently increased terrein production in two test strains (Yao et al., 2023). An additional effect of HstD was also proposed, namely that it could perform post-translational modifications of downstream regulators or enzymes, possibly influencing secondary metabolism on an additional level (Yao et al., 2023).

## 5 Similar gene clusters in other organisms

Several other, sometimes only distantly related organisms have been shown to possess a gene cluster similar to the *A. terreus ter* BGC. For the current work, we define “similar BGCs” as clusters that contain at least three genes homologous to any three genes of the *ter* BGC, with homology understood as a possible common evolutionary origin inferred from sequence similarity. It should be emphasized, however, that sequence similarity does not necessarily imply conservation of function. Nevertheless, incorporating these clusters into comparative studies has the potential to facilitate the functional characterization of genes within the *ter* BGC (Kahlert et al., 2021a).

*Aspergillus lentulus* has been shown to produce terrein, and accordingly, possesses a functional *ter* BGC, consisting of eleven genes (Takahashi et al., 2021). The overall structure, including gene content and gene orientation are almost identical to the BGC in *A. terreus* (Kahlert et al., 2021a; Takahashi et al., 2021). It was suggested that the regulation of the clusters in the two species is also similar (Takahashi et al., 2021).

The *CU-PKS-4* gene cluster (**Figure 1**) of the lichen forming fungus *Cladonia uncialis* encodes a polyketide synthase, a ketoreductase-dehydratase and a monooxygenase, homologues of TerA, TerB and TerD (Abdel-Hameed et al., 2016). The other two genes encoding a halogenase and an *O*-methyltransferase are seemingly not homologous to any of the genes in the *ter* BGC. It was suggested that the fungus synthesizes 6-hydroxymellein, and after several enzymatic steps, an oxidized, methylated, and halogenated derivative of 6-hydroxymellein is produced (Abdel-Hameed et al., 2016).

*Roussoella* sp. DLM33 produces a double chlorinated lactone and roussoellatide (a cryptosporiopsin-derived SM) among other SMs. In the genome of the fungus, a BGC similar to the *ter* BGC was identified (Kahlert et al., 2021a). The core genes are *RslA* and *RslB* which are homologs of *terA* and *terB*. Furthermore, a gene called *RslC* is homologous to *terC*, and a transcriptional activator denoted *RslR* is homologous to *terR* (Kahlert et al., 2021a). Additional genes in the *Roussoella* sp. DLM33 BGC encode two flavin-dependent halogenases (denoted *RslK* and *RslN*); a short-chain dehydrogenase/reductase (*RslO*) and an *O*-methyltransferase (*RslP*) (Kahlert et al., 2021a).

In a common root colonizing fungus (Mandyam et al., 2010; Knapp et al., 2012), *Periconia macrospinosa*, a putative cryptosporiopsinol BGC was identified, consisting of ten genes. These include homologues of *terA*-*B-C* and *terR*. Additionally, oxygenases, halogenases and an *O*-methyltransferase are predicted in the BGC, seemingly not being homologous to any of the *A. terreus ter* BGC genes but having similar genes in the *Roussoella* sp. DLM33 BGC (Kahlert et al., 2021a). *Periconia macrospinosa* is known to produce several chlorinated melleins (3,4-dihydroisocoumarins) and cyclopericodiol (Inose et al., 2019), which is a SM related to terrein. However, terrein synthesis was not verified in the fungus.

The fungus *Helminthosporium velutinum* produces cyclohelminthols (Honmura et al., 2016), SMs related to terrein (Ugai et al., 2020). Correspondingly, its genome contains a cluster (**Figure 1**), termed cyclohelminthol (*chm*) BGC (Ugai et al., 2020). The cluster contains nine genes, including *chmA*, *chmB* and *chmC*, homologues of *terA*-*B-C* and two putative halogenases, not showing homology to any of the genes of *ter* BGC (Ugai et al., 2020).

*Lachnum palmae* is a producer of palmaenones, which are cyclopentenones similar to terrein (Matsumoto et al., 2011). The palmaenone (*plo*) BGC (**Figure 1**) was identified in its genome as the one responsible for the synthesis of these SMs (Ugai et al., 2020). In the *plo* BGC five genes are encoded, and a distinct gene, located in another locus, also showed homology to a gene in the *chm* BGC. Homologues of *terA-B-C*, termed *ploA-B-C*, and three genes, among them two halogenases constitute the *plo* BGC (Ugai et al., 2020).

It is worth noting that in each of the above-described clusters, the core genes, that is the homologues of *terA* and *terB*, are encoded in opposite directions, as in the *ter* BGC in *A. terreus*, implying the possibility of conservation in the regulation of gene expression.

These data show that both closely and distantly related fungi harbor biosynthetic gene clusters that are similar to the *A. terreus ter* BGC, containing at least some putatively homologous genes. However, the chemical outputs and the exact functions often diverge. These *ter* BGC-like clusters differ in both size and complexity, ranging from smaller clusters, to the nearly identical BGC in *A. lentulus*, and to clusters that contain additional enzymes allowing more elaborate downstream modifications, in lichen-forming, plant-associated, or endophytic fungi. These organisms produce terrein-like or mellein-derived metabolites, supporting a conserved core biosynthetic logic with lineage-specific modifications.

## 6 Conclusions and outlook

Studies on the *ter* BGC have yielded several insights that may extend beyond *A. terreus* and contribute to the broader understanding of fungal secondary metabolism. Detailed investigations on the *ter* cluster (and similar BGCs in other organisms) have demonstrated that a relatively compact set of genes can produce a structurally complex polyketide through sequential tailoring reactions (Stroe et al., 2024). This supports the concept that such gene clusters represent versatile metabolic units in fungi. Moreover, studies suggesting that some *ter* genes are dispensable for terrein biosynthesis underline that BGCs may encode additional, accessory genes (Keller, 2019).

Functional analysis of the cluster-specific transcription factor TerR, together with its identified binding motifs, fits well into the established paradigm of transcriptional regulation in fungal secondary metabolism. TerR-dependent, direct transcriptional regulation, together with indirect transcriptional and chromatin-level modulation, illustrates how pathway-specific and epigenetic factors jointly determine metabolite output—a principle that appears to apply broadly to other fungal BGCs (Lyu et al., 2020). The presence of additional layers of regulation, including global transcription factors responding to environmental signals, and pathway crosstalk, further reinforces the concept of multi-layered regulatory networks controlling fungal secondary metabolite gene clusters (Kwon et al., 2021).

The product of the collaborative action of TerA and TerB (and homologous proteins), 6-hydroxymellein (Kahlert et al., 2021b), seems to be a key intermediate in the synthesis of terrein (Kahlert et al., 2021a) and related SMs (Ugai et al., 2020). Indeed, several terrein derivatives are known in fungi (Reveglia et al., 2020), such as cryptosporiopsin, palmaenones, and others (Ugai et al., 2020; Kahlert et al., 2021a). Notably, the *ter* BGC lacks halogenases and *O-*methyltransferases when compared to the similar clusters mentioned above. Instead, *A. terreus* uses oxidases in the subsequent steps of the synthesis of terrein (**Figure 1**). The presence of halogenases and *O-*methyltransferases allows the formation of a wide range of chlorinated and methylated derivatives, such as 5,7-dichloro-6-hydroxymellein, cryptosporiopsin and roussoellatide, among others, which are not produced in *A. terreus*.

While many functions of terrein have been identified, its general ecological significance is less clear. The phytotoxic activity of terrein - causing lesions on fruit surfaces and inhibiting seed germination - may facilitate plant host colonization or opportunistic pathogenic interactions with plant hosts. The antibacterial and antifungal activities of terrein may aid competition against co-occurring microbes in shared habitats. Additionally, the function of terrein as a reductive agent that mobilizes iron suggests an important contribution to nutrient acquisition under iron-limited conditions. Together, these properties imply that terrein could have multiple ecological roles. Despite the progress made in elucidating the structure, functions, and regulation of the *ter* BGC, substantial gaps in knowledge remain. The precise roles of several cluster-encoded proteins are still only partially understood or entirely unknown (Yin et al., 2016). The reason for this is that the isolation and structure elucidation of metabolites from different KO transformants lacking *ter* genes is often unsuccessful because of their instability and/or reactivity (Zaehle et al., 2014). The functional characterization is also hampered by other technical difficulties, such as the low yields and incompatibility of enzymes in heterologous expression systems (Kahlert et al., 2021a).

This represents a limitation in our understanding of terrein biosynthesis in *A. terreus*. The functions could, theoretically, be elucidated using a combination of approaches. These include (i) targeted genetics, such as gene knockouts, overexpression studies, optimal expression of individual genes or combinations of them in the native host or in an optimal heterologous system (Kjærbølling et al., 2019; Lin et al., 2020), (ii) *in vitro* enzyme experiments (Cacho and Tang, 2016; Kahlert et al., 2021a) (iii) stable isotope labeling and precursor feeding experiments (Zaehle et al., 2014; Kahlert et al., 2021a).

Moreover, while pathway-specific and certain global regulators of terrein biosynthesis have been characterized, the upstream signaling cascades and the extent of regulatory crosstalk with other signaling pathways have yet to be explored. Epigenetic influences on terrein production have only been partially explored, and their overall contribution remains unclear.

Finally, the ecological role and the possible adaptive significance of similar BGCs in other organisms is poorly understood. Resolving these open questions will be essential for both advancing basic fungal biology and may support exploring potential applications of fungal SMs.

A deeper understanding of the terrein biosynthetic pathway and its regulatory network could also enable a range of biotechnological applications, such as metabolic engineering for enhanced terrein production, production of terrein derivatives (possibly with bioactivities), and the utilization of individual tailoring enzymes for synthetic biology or for the synthesis of custom-designed derivatives.
